# Amphibian Beta Diversity in the Brazilian Atlantic Forest: Contrasting the Roles of Historical Events and Contemporary Conditions at Different Spatial Scales

**DOI:** 10.1371/journal.pone.0109642

**Published:** 2014-10-08

**Authors:** Fernando Rodrigues da Silva, Mário Almeida-Neto, Mariana Victorino Nicolosi Arena

**Affiliations:** 1 Departamento de Ciências Ambientais, Universidade Federal de São Carlos, Sorocaba, São Paulo, Brazil; 2 Departamento de Ecologia, Universidade Federal de Goiás, Goiânia, Goiás, Brazil; University of Brasilia, Brazil

## Abstract

Current patterns of biodiversity distribution result from a combination of historical and contemporary processes. Here, we compiled checklists of amphibian species to assess the roles of long-term climate stability (Quaternary oscillations), contemporary environmental gradients and geographical distance as determinants of change in amphibian taxonomic and phylogenetic composition in the Brazilian Atlantic Forest. We calculated beta diversity as both variation in species composition (CBD) and phylogenetic differentiation (PBD) among the assemblages. In both cases, overall beta diversity was partitioned into two basic components: species replacement and difference in species richness. Our results suggest that the CBD and PBD of amphibians are determined by spatial turnover. Geographical distance, current environmental gradients and long-term climatic conditions were complementary predictors of the variation in CBD and PBD of amphibian species. Furthermore, the turnover components between sites from different regions and between sites within the stable region were greater than between sites within the unstable region. On the other hand, the proportion of beta-diversity due to species richness difference for both CBD and PBD was higher between sites in the unstable region than between sites in the stable region. The high turnover components from CBD and PBD between sites in unstable *vs* stable regions suggest that these distinct regions have different biogeographic histories. Sites in the stable region shared distinct clades that might have led to greater diversity, whereas sites in the unstable region shared close relatives. Taken together, these results indicate that speciation, environmental filtering and limited dispersal are complementary drivers of beta-diversity of amphibian assemblages in the Brazilian Atlantic Forest.

## Introduction

Current patterns of biodiversity distribution result from a combination of historical and contemporary processes [1]–[2]. As a consequence, we could expect that the more current and past environmental conditions differ from each other, the less predictive models based on present-day variables will be. In order to understand the extent to which past conditions have affected current biodiversity distribution, several studies have contrasted the roles of historical processes and current environmental conditions in order to understand patterns of species distribution [3]–[6]. According to climate-diversity hypotheses, climatic variables that reflect present-day conditions are the key drivers of speciation, extinction and dispersal rates, thus also influencing current patterns of species distribution [7]–[8]. For example, distinct climatic conditions between habitats or regions might restrict the dispersal of individuals within the distribution limits of ancestral species - niche conservatism [7], or might promote the extinction of some species due to differences in physiological tolerance - physiological tolerance hypothesis [9]. In contrast, the historical hypothesis postulates that the duration and extent of stable climatic conditions in Earth's history have allowed more opportunity for diversification due to high speciation and/or low extinction rates [4], [6]. Furthermore, many studies have demonstrated that dispersal from the center of origin or from past refugia also play a key role in explaining current patterns of species distribution for many phylogenetic lineages [10]–[11]. According to Araújo *et al.* [6], narrow-ranging species of reptiles and amphibians occur preferentially in areas not covered by ice during the last glacial period in Europe, suggesting that low colonization ability limited the geographical distribution of these species during the interglacial period.

A practical challenge in comparing the effects of historical versus current conditions on the distribution of species diversity is to determine which components of diversity should reflect each process. Studies integrating compositional beta diversity (generally defined as variation in species composition among sites; hereafter CBD) and phylogenetic beta diversity (defined as the amount of shared phylogenetic history between two communities; hereafter PBD) have provided different insights into the ecological and evolutionary mechanisms that structure communities [12]–[15]. These frameworks allow us to evaluate how ecological processes, such as environmental filtering and dispersal, interact with historical processes (speciation and extinction), influencing extant patterns of distribution within and among different regions. For instance, high CBD together with low PBD indicates a high proportion of small-ranged species, reflecting speciation across regions [16]. In contrast, high CBD together with high PBD indicates low lineage dispersal across regions [16]. However, studies combining CBD and PBD to identify the lineages that are driving the patterns of turnover between regions are still scarce [5], [12]–[15]. In order to test the relative importance of historical events and current environmental gradients, we investigated the spatial distribution of CBD and PBD on amphibians within the Atlantic Forest, a forest biome where humid forests retracted during the cooler, drier period of the Quaternary's climate oscillations without being covered by ice [17].

The Atlantic Forest is a highly threatened global biodiversity hotspot [18], with more than 500 known amphibian species, of which 88% are endemic [19]. The Pleistocene refuge hypothesis [20] suggests that during the cold dry conditions of the Last Glacial Maximum (LGM), approximately 21 000 yr BP, some areas (i.e. Pernambuco refuge, Bahia refuge and Southeastern refuge) in the Atlantic Forest experienced less variability in temperature and precipitation [17], [21], [22]. These areas not only served as a large climatic refugium for Neotropical species but also promoted local evolutionary differentiation and diversification, allowing for greater species turnover [22]–[25]. Based on this scenario, recent studies have modeled the spatial range of the Brazilian Atlantic Forest for different climatic scenarios (current and past) to examine whether the regions predicted to have remained stable across climatic fluctuations were consistent with current patterns of species endemism in coastal Brazil [21], as well as genetic diversity for certain populations [23]–[24]. However, these studies did not examine the current patterns of species diversity encompassing contemporary environmental conditions and different spatial extents among regions and local communities in this biome.

We compiled data on amphibian species composition to develop a thorough understanding of the processes driving the CBD and PBD patterns of amphibian distribution in the southern range of the Brazilian Atlantic Forest. First, we partitioned CBD and PBD into two distinct components: spatial turnover and nestedness. The partition of total beta diversity allowed us to infer the different processes (replacement or gain/loss of species) that structure communities [26]–[28]. Then, we assessed the relative roles of current environmental gradients, geographic isolation and long-term climatic conditions (Quaternary climatic oscillation) in shaping the present-day patterns of CBD and PBD. Specifically, we expected that the relative proportion of amphibian CBD and PBD that is due to species loss (extinction or limited dispersal) should be higher in areas where the effects of Quaternary climate changes were stronger (unstable regions), whereas the relative proportion of amphibian CBD and PBD that is due to spatial replacement (speciation) should be higher in areas that served as a large climatic refugium for Neotropical species (stable regions). Furthermore, based on physiological constraints and limited dispersal, two key characteristics of amphibians, we expected that variations in precipitation, temperature and/or altitude would be the environmental conditions that constrained the similarities in species composition.

## Materials and Methods

### Study area and climate data

We used checklists of local amphibian communities from 44 sites along the southern range of the Brazilian Atlantic Forest (see Appendix S1 in File S1 and Data S1). Because we obtained all data from literature surveys, no specific permissions were required. We limited our study to the southern range of the Brazilian Atlantic Forest because checklists of amphibians do not equally cover all the extent of the Atlantic Forest. The southeastern region is relatively over-represented while the northern and the extreme southern regions are under-represented. To reduce possible biases due to methodological differences in sampling procedures, we selected only sites whose checklists met the following criteria: (i) at least two out of four different survey methodologies (audio, active search, casual observations, and pitfall traps); (ii) samplings in all seasons for at least one year (Appendix S1 in File S1). Furthermore, anuran species not identified (e.g., *Leptodactylus* sp.) were excluded from the analysis. Although we recognize the difficulties posed by studies considering data from different inventories, we maintain that the above-cited criteria provide a more reliable checklist than geographic range maps [29].

We used the WorldClim data base [30] at a resolution of 2.5′ and DIVA-GIS 7.5 [31] to obtain the following climatic variables for each forest site: (1) annual mean temperature (ANNT); (2) maximum temperature of the warmest month (MAXT); (3) minimum temperature of the coldest month (MINT); (4) difference between MAXT and MINT (DIFT); (5) annual precipitation (PPT); (6) precipitation seasonality (coefficient of variation across months) (PPTS); (7) precipitation of wettest quarter (PPTW); (8) precipitation of driest quarter (PPTD); and (9) difference between PPTW and PPTD (DIFP). Furthermore, we used Google Earth to obtain the following topographical data: (10) maximum elevation (MAEL); (11) minimum elevation (MIEL); and (12) elevational range (difference between MAEL and MIEL: DIEL). These variables were used because they describe the average trends as well as variation in temperature, precipitation and elevational range which might represent physiological limits for amphibians [32]–[34].

### Data analysis

#### Compositional Beta Diversity (CBD)

We used two additive partitioning frameworks proposed by Baselga [26]–[27] and Carvalho *et al.* [35] to determine the CBD. Although these approaches are intended to measure species replacement and species richness differences, their methods can lead to radically different conclusions using the same dataset [see 36 for comparisons of these methods]. In short, both Baselga [26]–[27] and Carvalho *et al.*'s [35] approaches consist of decomposing the pair-wise Jaccard dissimilarity index (β_jac_ and β_cc_ respectively) into two additive components. Specifically, Baselga's [27] approach consists of: i) the turnover component (β_jtu_), which measures the proportion of unique species in two sites pooled together if both sites are equally rich, and ii) the nestedness-resultant component (β_jne_), which measures how dissimilar the sites are due to a nested pattern. Carvalho *et al.*'s [35] approach consists of: i) species replacement (β_-3_), which describes a species at one site that is substituted by a species at another site, and ii) richness disparities (β_rich_), which reflect the absolute difference between the number of species that each site contains, irrespective of nestedness. In this study, Baselga and Carvalho *et al.*'s approaches produced qualitatively similar results. Therefore, we will present only the results from Baselga's approach (see Appendix S2 in File S1 for a discussion about the approaches).

#### Phylogenetic Beta Diversity (PBD)

To assess the phylogenetic similarities among all species in our dataset, we constructed a cladogram based on the time-calibrated tree proposed by Pyron & Wiens [37] (Appendix S3 in File S1), which contains 2,871 species (40% of known extant species) from 432 genera (85% of the 500 currently recognized extant genera). We pruned the time-calibrated tree to include only the anuran species found on 44 sites used in this study. Only seven out of the 50 recorded genera (*Zachaenus*, *Megaelosia*, *Arcomover*, *Crossodactylodes*, *Myersiella*, *Stereocyclops* and *Euparkerella*; Appendix S4 in File S1) were not present on the time-calibrated tree of Pyron & Wiens [37]. Thus, the anuran species whose genera were present in the tree were inserted as within-genus polytomies while the species belonging to the genera not present in the tree were inserted based on phylogenetic relationships from other sources [38]–[39]. We acknowledge that polytomies under-sample branch length differences among species. However, polytomies are generally more sensitive to loss of resolution basally in the phylogeny and less sensitive to loss of resolution terminally [40], as represented in this study.

We quantified phylogenetic dissimilarities using the UniFrac index [41]. Values of UniFrac range from 0 (indicating that the two communities are composed of similar species) to 1 (indicating that the two communities are composed of distinct species). Like the beta diversity index, the UniFrac index has recently been decomposed into two components representing “true” phylogenetic turnover (UniFrac_Turn_) and phylogenetic diversity gradients (UniFrac_PD_) [28]. According to Leprieur *et al*. [28], UniFrac_Turn_ measures the relative amount of gains and losses of unique lineages between communities that is not attributable to their differences in phylogenetic diversity. In contrast, UniFrac_PD_ measures the amount of phylobetadiversity caused by differences in phylogenetic diversity between phylogenetically nested communities. Following Leprieur *et al.* [28], we tested whether pairs of assemblages were more or less phylogenetically dissimilar than expected by chance, using a null model in which species richness and the CBD between regions were fixed and only the identities of the species in the phylogeny were randomized 999 times. A standardized effect size (SES) was calculated for PBD and its components. SES values greater than 1.96 indicate a higher PBD than expected by CBD, while SES values below −1.96 indicate a lower PBD than expected by CBD [28]. If observed values of PBD do not differ from what would be expected by chance alone, then PBD is unlikely to be the result of historical processes.

#### Delimitation of stable and unstable regions

**Figure 1:**
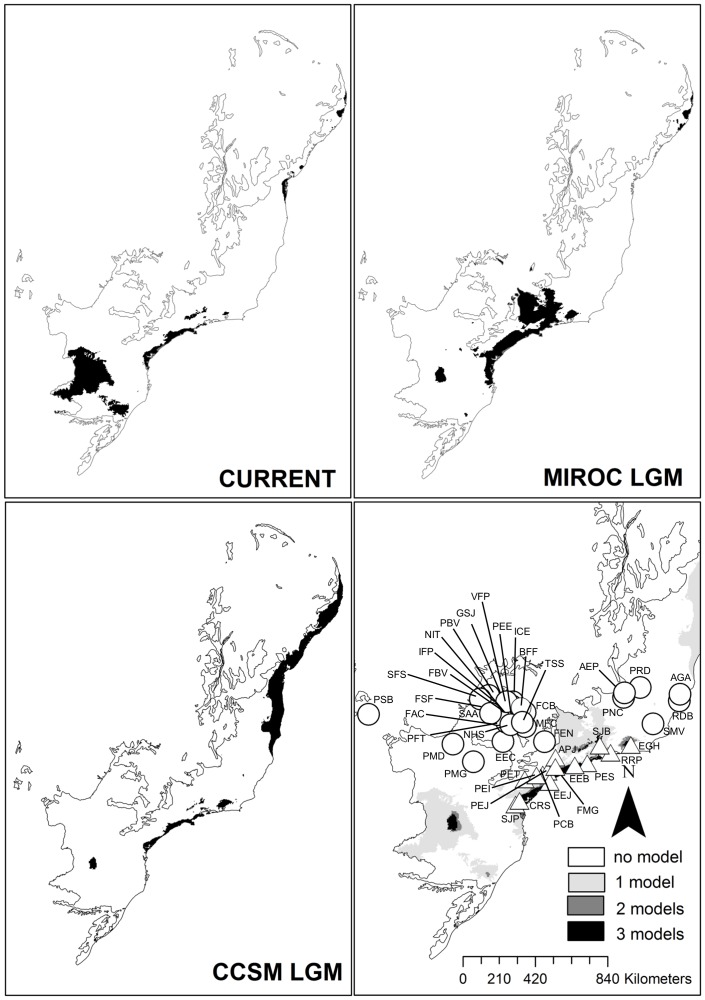
Distribution of the10% highest values of annual precipitation for the current time, the two last glacial maximum period (LGM), and the intersection of the three maps. Symbols (in the intersection map) indicate the 44 sites in the southern range of Brazilian Atlantic Forest. Dark shading indicates stable regions. White circles represent sites in an unstable region (Quaternary Climatic Oscillations). White triangles represent sites in a stable region (Quaternary Climatic Stability). Abbreviations of each site as in Appendix S1 in File S1.

The major processes that control the distribution of species diversity may vary depending on the spatial or temporal extent of the study system [42] and the regional species pool [43]. To delimit the spatial extent of stable and unstable areas, we projected the annual precipitation distribution of the current time period onto two paleoclimate scenarios simulating the last glacial maximum period (LGM) 21,000 years ago: CCSM3 (Community Climate System Model, available at: http://www.worldclim.org/past) and MIROC (Model of Interdisciplinary Research on Climate, available at: http://www.worldclim.org/past) at a resolution of 2.5′ [30]. Then we produced a binary map by transforming 10% of grids with the highest values of annual precipitation into one and the remaining grids to zero. For last, we defined the stable climatic areas as intersections on maps for which the highest values of annual precipitation were inferred in all models (Fig. 1). The intersection map shows a coastal area of long-term climatic stability (Fig. 1). Therefore, the spatially explicit predictions of climatic distribution in the southern range of the Atlantic Forest are consistent with the refugia areas described in other studies [22]–[24]. These data were used as predictor variables (unstable and stable regions) in the subsequent analyses.

#### Relative importance of geographical distance, current environmental gradients and long-term climatic conditions in explaining variations in CBD and PBD components

In order to reduce the data dimensionality and multicollinearity, we performed a principal component analysis (PCA) based on a correlation matrix of the data considering temperature variables (ANNT, MAXT, MINT and DIFT), precipitation variables (PPT, PPTS, PPTW, PPTD and DIFP) and elevation-related variables (MAEL, MIEL and DIEL). The first three axes of the principal component explained 85% of the variations in the climatic and topographic data (Appendix S5 in File S1). Therefore, for the subsequent analysis we calculated for each pair of sites the Euclidean distance based on all three PC axes - current environmental gradients [44].

The relative importance of current environmental gradients, geographical distance (Euclidean distance inferring the distance decay of similarity between sites [45]) and long-term climatic conditions in explaining variations in CBD and PBD components was examined using a variance partitioning technique where the total percentage of the variation of ordinary least-squares regressions is partitioned into unique and common contributions of the sets of predictors [46]. The total variation of CBD and PBD was divided into eight fractions: 1) variation explained purely by environmental gradients; 2) variation explained purely by geographical distance; 3) variation explained purely by long-term climatic conditions; 4) variation explained by environmental gradients and geographical distance together; 5) variation explained by environmental gradients and long-term climatic conditions together; 6) variation explained by geographical distance and long-term climatic conditions together; 7) variation explained by environmental gradients, geographical distance and long-term climatic conditions together; and 8) unexplained (residual). For these analyses, current environmental gradients and geographical distance were log-transformed.

All analyses were conducted in R 3.1.0 [47] using betapart [48], vegan [49] and ape [50] packages available at http://www.r-project.org/.

## Results

### Partitioning of CBD and PBD

**Figure 2:**
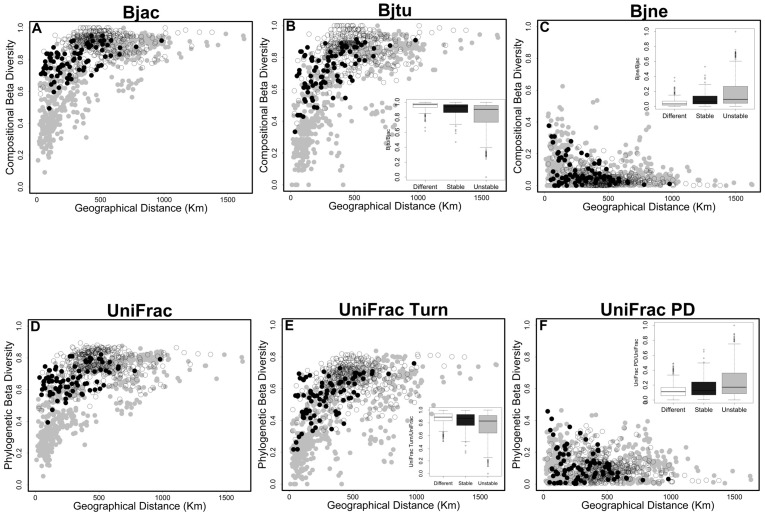
Relationships between geographical distance and Compositional Beta Diversity (CBD, A = β_jac_, B = β_jtu_ and C = β_jne_) and Phylogenetic Beta Diversity (PBD, D = UniFrac_Total_, E = UniFrac_Turn_ and F = UniFrac_PD_) components. Subplots are showing the proportion explained by turnover (β_jtu_/β_jac_ and UniFrac_Turn_/UniFrac_Total_) and nestedness (β_jne_/β_jac_ and UniFrac_PD_/UniFrac_Total_) components. Symbol colors indicate the region where the sites occur in the southern range of Brazilian Atlantic Forest. Black circles indicate dissimilarity between sites within stable region (Quaternary Climatic Stability). Gray circles indicate dissimilarity between sites within unstable region (Quaternary Climatic Oscillations). White circles indicate dissimilarity between unstable and stable sites (different regions).

**Figure 3:**
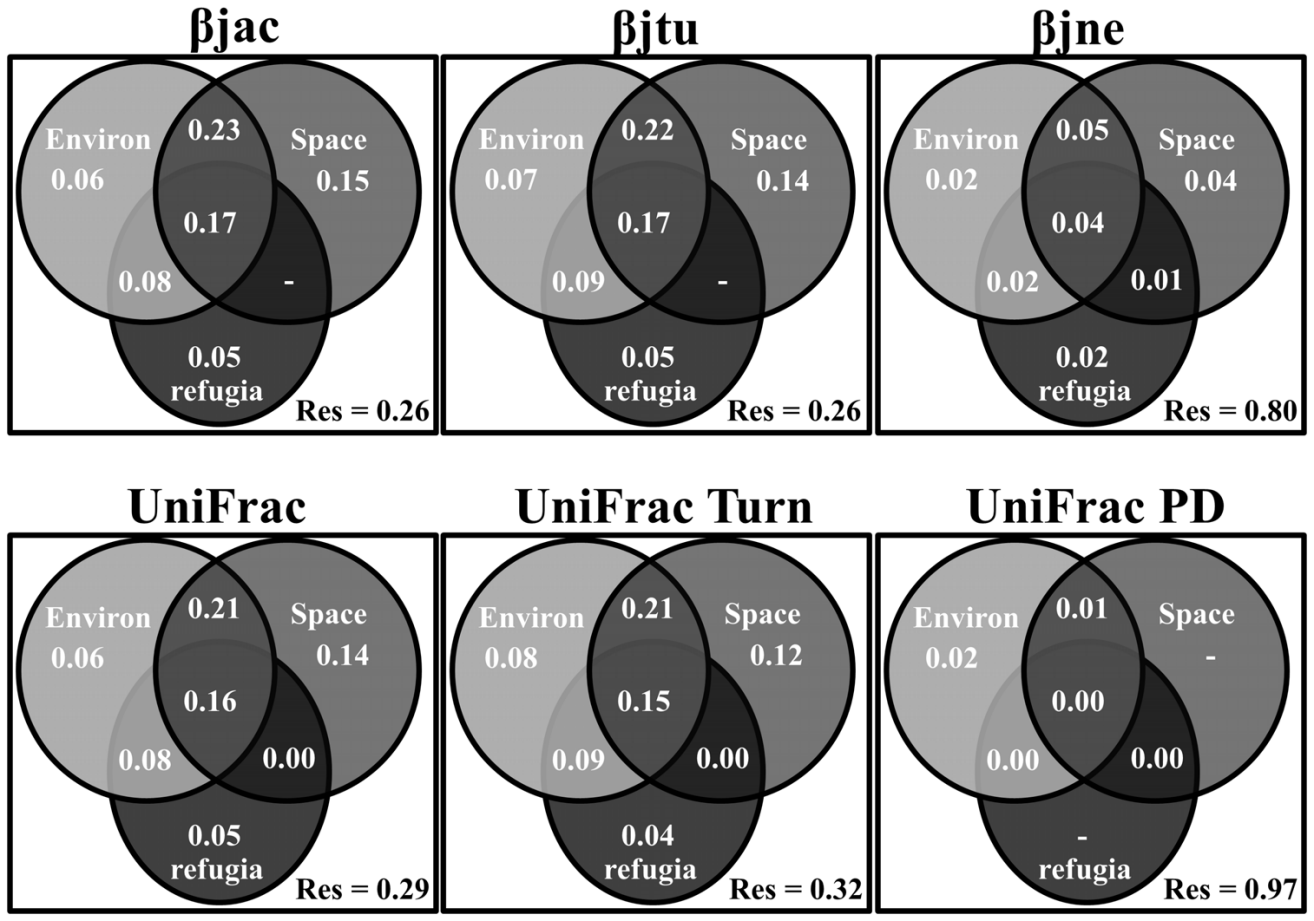
Partition of the variance of Compositional Beta Diversity (CBD, β_jac_, β_jtu_ and β_jne_) and Phylogenetic Beta Diversity (PBD, UniFrac_Total_, UniFrac_Turn_ and UniFrac_PD_) components explained by geographical distance (Space), current environmental gradients (Environ) and long-term climatic conditions (refugia) for 44 sites in the southern range of Brazilian Atlantic Forest. Res  =  unexplained variance. “-”  =  variation explained <0.

**Figure 4:**
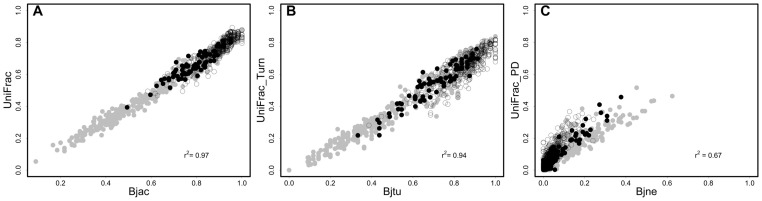
Relationship among Compositional Beta Diversity (CBD, A  =  β_jac_, B  =  β_jtu_ and C  =  β_jne_) and Phylogenetic Beta Diversity (PBD, A  =  UniFrac_Total_, B  =  UniFrac_Turn_ and C  =  UniFrac_PD_) components. Symbol colors indicate the region where the sites occur in the Brazilian Atlantic Forest. Black circles indicate dissimilarity between sites within stable region (Quaternary Climatic Stability). Gray circles indicate dissimilarity between sites within unstable region (Quaternary Climatic Oscillations). White circles indicate dissimilarity between unstable and stable sites (different regions).

A total of 238 amphibian species (Appendix S4 in File S1) were recorded in the 44 sites. The stable (14 sites) and unstable (30 sites) regions harbored 160 and 150 amphibian species, respectively, and only 71 amphibian species (29.8%) occurred in both regions. The partitioning of CBD and PBD revealed that the turnover component was the major reason for amphibian dissimilarity among sites (Fig. 2). Current environmental gradients and geographical distance together and these two variables with long-term climatic conditions explained on average about 25% and 21%, respectively, of the variation in turnover components for both CBD and PBD (Fig. 3). In fact, turnover components of CBD and PBD were highly correlated (Fig. 4), indicating that variation in PBD is explained by the turnover of CBD, or vice-versa. On the other hand, current environmental gradients, geographical distance and long-term climatic conditions explained a small proportion of the total variation of nestedness (β_jne_–20%) and phylogenetic diversity (UniFrac_PD_–2%) (Fig. 3).

**Figure 5:**
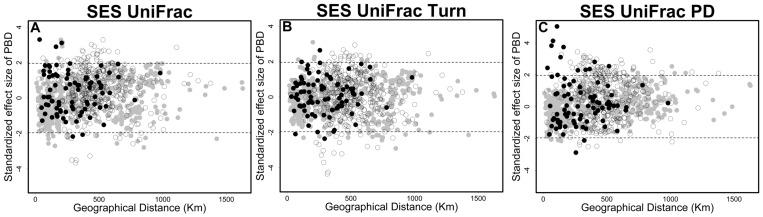
Standardized effect size (SES) values for phylogenetic beta diversity (PBD, A  =  UniFrac_Total_, B  =  UniFrac_Turn_ and C  =  UniFrac_PD_) components among 44 sites in Brazilian Atlantic Forest. Values between dashed lines indicate that PBD components have no difference with respect to null expectation. Symbol colors indicate the region where the sites occur in the southern range of Brazilian Atlantic Forest. Black circles indicate SES between sites within stable region (Quaternary Climatic Stability). Gray circles indicates SES between sites within unstable region (Quaternary Climatic Oscillations). White circles indicate SES between unstable and stable sites (different regions).

We observed a positive relationship between the geographical distance and turnover components of CBD and PBD (Fig. 2). Values of β_jtu_ and UniFrac_Turn_ between sites in different regions or between sites within stable region were on average 0.25 and 0.16 higher, respectively, than between sites within unstable region (Fig. 2). Although the PBD pattern may be explained by species dissimilarities between sites, some UniFrac_Turn_ values between sites in different regions were higher than expected by the null expectation (Fig. 5), indicating the influence of historic processes. On the other hand, a negative relationship was observed between values of β_jne_ and UniFrac_PD_ and geographical distance (Fig. 2). The average proportion of β_jne_ values between sites in different regions was 0.03, showing that CBD between sites in different regions are not subsets of each other. As expected, the proportion of nestedness components for both CBD and PBD were higher between sites in unstable region than between sites in stable region (Fig. 2). Furthermore, only some UniFrac_PD_ values between sites in different regions and within stable region were higher than expected by the null expectation (Fig. 5), indicating that species composition in sites within unstable region are from the same lineages (i.e. same genera).

## Discussion

Our results show that patterns of amphibian CBD and PBD in the southern range of Brazilian Atlantic Forest were largely determined by lineage turnover. Interestingly, the variance in turnover components from CBD and PBD is equally explained by current environmental gradients, geographical distance and long-term climatic conditions. However, the turnover components between sites from different regions and between sites within stable region were greater than between sites within unstable region. These results are consistent with previous studies that stressed that patterns of species distribution are influenced by different processes depending on the regional species pool [42], [51] and the spatial scale [52] considered.

Historical effects, environmental filtering and limited dispersal are recognized processes influencing the spatial distribution of turnover components of CBD and PBD among communities, and, consequently, determining which lineages reside in a particular region [16], [42], [53]–[54]. Even though stable and unstable regions are adjacent to each other, the high turnover components from CBD and PBD between sites from these distinct areas suggest that stable and unstable regions have different biogeographic histories. For example, eleven genera (*Arcovomer*, *Brachycephalus*, *Cycloramphus*, *Gastrotheca*, *Holoaden*, *Macrogenioglosus*, *Megaelosia*, *Myersiella*, *Paratelmatobius*, *Phrynomedusa* and *Scythrophrys*) were only recorded in sites within the stable region while nine genera (*Ameerega*, *Crossodactylodes*, *Dermatonotus*, *Eupemphix*, *Melanophryniscus*, *Phyllodytes*, *Pseudis Pseudopaludicola* and *Stereocyclops*) were only recorded in sites within the unstable region (Appendix S4 in File S1). According to Jansson [55], the higher is the long-term climatic stability in an area, the more new clades will persist without going extinct or reuniting with other clades. These results suggest that sites in stable regions may be areas of high diversification because of ameliorate conditions promoting high speciation and/or low extinction rates of anuran species with specialized reproductive modes (e.g. direct development of terrestrial eggs). Furthermore, current environmental gradients, temperature and precipitation in particular (see Appendix S4 in File S1), seem to act as filters to disperse species from stable (area of origin) to unstable regions. This result is similar to that found by da Silva *et al.* [34], who showed that moister sites in the Atlantic Forest harbored a greater phylogenetic diversity of amphibians than drier sites. Therefore, the interplay among historical effects, environmental filtering, and dispersal limitations might have contributed to the high values of turnover between sites in unstable and stable regions for both CBD and PBD. These results are in agreement with other studies showing that amphibian beta diversity is influenced by multiple factors [6], [56]–[58]. For example, broad-scale amphibian richness is strongly determined by historical constraints whereas regional patterns are determined by water and temperature [33].

At the regional scale, turnover components between sites within the stable region are larger than corresponding values between sites within the unstable region (Fig. 4). This result demonstrates that, although sites in stable region are relatively close, there are other factors driving CBD and PBD between them. Deviances from the expected distance-decay relationship usually happen if the distances are associated with marked geographical barriers to dispersal [15]. It is well-known that larger elevational ranges promote speciation through habitat specialization and isolation, thus increasing endemism and, consequently, discrepancies in species richness between sites within a region [59]–[64]. Sites in stable regions are situated in the coastal Atlantic Forest that harbors the mountain complex of Serra do Mar and Serra da Mantiqueira, which may act as barriers to the dispersion of amphibians. Kozak & Wiens [65] showed that niche conservatism is the main factor promoting allopatric speciation and endemism in montane salamanders. According to these authors, allopatric sister taxa inhabiting similar climatic niches are not able to cross lowlands, which seemingly leads to geographic range fragmentation and speciation. Graham et al. [12] also found that the mountains are major dispersal barriers to hummingbird species, creating large differences in species composition despite the communities being phylogenetically similar. Therefore, these results suggest that topographic conditions and dispersal limitations can interact with evolutionary processes, influencing greater turnover components of CBD and PBD between sites within stable regions more than in unstable regions, usually associated to lowlands, within the Atlantic Forest.

As expected, the proportion of nestedness components from CBD was greater between sites in unstable rather than stable regions. This finding is similar to those reported by studies analyzing broad-scale effects of Quaternary climate conditions. For example, on a global scale, differences in fish faunas characterized by nestedness were greater in drainage basins that experienced larger amplitudes of Quaternary climate oscillations [66]. At the continental scale, beta diversity patterns at high latitudes of amphibians, birds and mammals from the New World were mostly determined by species richness differences in areas that were affected by glaciation until recently [57]. On the other hand, PBD between stable and unstable sites were not phylogenetically nested. Because nestedness can only occur between communities from the same overall species pool, the low values of PBD strengthened the idea that stable and unstable regions experience different biogeographic histories. Taken together, our results reinforce previous studies that highlight the importance of considering beta diversity components for CBD and PBD across multiple scales to infer how current environmental gradients and historical factors have influenced speciation, extinction and the dispersal of species throughout a region [12], [14], [16], [28], [58].

## Conclusion

Our results show that the relative roles of geographical distance, current environmental gradients and long-term climatic conditions in explaining the variations of amphibian CBD and PBD depend on the spatial scale under consideration. According to Belmaker & Jetz [52], the spatial scale at which environmental conditions constrain species richness will differ across clades with different home ranges and dispersal abilities. Therefore, this study demonstrates that historical events, current climatic conditions and geographical distances are complementary predictors of amphibian composition even for sites within the same biome. These results are in accordance with Buckley & Jetz [33], who analyzed the distribution of amphibians on a global scale and highlighted the importance of considering both the environment and historical events when attempting to understand gradients of amphibian distribution.

Furthermore, the variation in CBD and PBD among the amphibian assemblages indicates that stable and unstable regions have different biogeographic histories. We observed the highest values of CBD and PBD between sites of different regions (unstable *vs* stable), indicating low lineage dispersal across regions [16]. These results are in accordance with macroecological studies considering the Atlantic Forest. For example, Villalobos et al. [67] and Loyola et al. [68] showed that stable regions considered in our study harbored higher numbers of amphibian species with small range sizes (i.e. high endemism) and phylogenetic diversity than unstable regions. The same pattern was observed by Araújo et al. [6] to amphibian species with narrow ranges in Europe that occurred preferentially in areas that remained favorable during the last glacial period. We also observed the lowest values of CBD and PBD between sites within unstable regions, indicating homogenization of taxonomic and phylogenetic composition within a region that harbors amphibian species with broad range sizes. Evidence from phylogeographic studies suggest that populations within unstable regions were only recently colonized via dispersal of migrants from sites within stable regions in southern areas of Atlantic Forest [23]–[24]. Based on this scenario, we suggest that current patterns of amphibian species distribution in the southern range of Brazilian Atlantic Forest may be determined by high speciation rates in long-term climatic stability and limited dispersion to other regions due to the mountain complex of Serra do Mar and Serra da Mantiqueira, and precipitation gradients that act as barriers to some amphibian species with specific life history traits [34], [69].

## Supporting Information

Data S1 **Presence and absence of anuran species in the 44 sites of Brazilian Atlantic Forest used in the analysis.**
(XLS)

File S1 Appendix S1, Description of 44 sites of Brazilian Atlantic Forest used in the analysis. Appendix S2, Partition of the variance of Compositional Beta Diversity components based on Carvalho's *et al.* (2012, β_cc_, β_3_ and β_rich_). Appendix S3, Cladogram of anuran demonstrating the phylogenetic relationships of our data-set based on the phylogenetic hypotheses proposed by Pyron & Wiens (2011). Appendix S4, Taxonomic classification and occurrence of amphibian species in stable and unstable sites from Brazilian Atlantic Forest. Appendix S5, Analysis summary of the principal component analysis (PCA) for 44 sites in the Brazilian Atlantic Forest.
(DOC)
