# Supplementary material for: Amphibian Beta Diversity in the Brazilian Atlantic Forest: Contrasting the Roles of Historical Events and Contemporary Conditions at Different Spatial Scales
Source: PLoS One. 2014 Oct 8;9(10):e109642. doi: 10.1371/journal.pone.0109642 (PMC4190317; doi:10.1371/journal.pone.0109642)
Supplement: File S1 — Appendix S1, Description of 44 sites of Brazilian Atlantic Forest used in the analysis. Appendix S2, Partition of the variance of Compositional Beta Diversity components based on Carvalho's et al. (2012, βcc, β3 and βrich). Appendix S3, Cladogram of anuran demonstrating the phylogenetic relationships of our data-set based on the phylogenetic hypotheses proposed by Pyron & Wiens (2011). Appendix S4, Taxonomic classification and occurrence of amphibian species in stable and unstable sites from Brazilian Atlantic Forest. Appendix S5, Analysis summary of the principal component analysis (PCA) for 44 sites in the Brazilian Atlantic Forest. (DOC) [file pone.0109642.s002.doc]

Appendix S1 - Description of 44 sites of Brazilian Atlantic Forest used in the analysis. Sampling methods: (1) Auditory; (2) active search; (3) casual meeting, and (4) pitfall traps.

|  | **Abbreviations** | **Latitude** | **Longitude** | **Species**  **Richness** | **Sampling efforts (days)** | **Sampling Period** | **Sampling**  **Methods** | **References** |
| --- | --- | --- | --- | --- | --- | --- | --- | --- |
| Parque Estadual Carlos Botelho | PCB | 24.112 | 47.627 | 64 | 12 | 2005 to 2006 | 1;3 | Bertoluci et al. 2007; Forlani et al. 2010 |
| Parque Estadual Alto do Ribeira | PET | 24.375 | 48.566 | 55 | 15 | 2009 | 1;2;3 | Araujo et al. 2010 |
| Reserva Biológica de Duas Bocas | RDB | 20.276 | 40.501 | 49 | 60 | 2007 to 2008 | 1;4 | Tonini et al. 2010 |
| Parque Estadual do Jurupará | PEJ | 23.976 | 47.376 | 45 | 32 | 2005 to 2007 | 1;2;3;4 | Condez et al. 2009 |
| Reserva Rio das Pedras | RRP | 22.99 | 44.089 | 41 | 82 | 1997 to 2006 | 1;2 | Carvalho-e-Silva et al. 2008 |
| Área de Proteção Ambiental Goiapava-Açu | AGA | 19.932 | 40.458 | 41 | * | * | 1;2 | Ramos & Gasparini 2004 |
| Campo Escoteiro Geraldo Hugo Nunes | EGH | 22.58 | 43.034 | 39 | * | 1983 to 2009 | 1;2 | Silva-Soares et al. 2010 |
| Parque Estadual da Serra do Mar | PES | 23.536 | 45.306 | 39 | 260 | 2000 to 2002 | 1;2;4^#^ | Hartmann 2004 |
| Reserva Particular Santuário do Caraça | PNC | 20.097 | 43.404 | 37 | 24 | 2001 to 2002 | 1 | Canelas & Bertoluci 2007 |
| Parque Estadual do Rio Doce | PRD | 19.586 | 42.538 | 36 | * | 1982 to 1997 | * | Feio et al. 1998 |
| Serro e Gemido -São José dos Pinhais | SJP | 25.683 | 49.05 | 34 | 17 | 2003 to 2004 | 1;2 | Conte & Rossa-Feres 2006 |
| Estação Ecológica Boracéia | EEB | 23.65 | 45.956 | 64 | * | * | 1;2 | Heyer et al. 1990 |
| São José do Barreiro | SJB | 22.619 | 44.653 | 32 | 60 | 2004 to 2006 | 1;2 | Serafim et al. 2008 |
| Condomínio Rio Sagrado | CRS | 25.498 | 48.841 | 31 | 19 | 2002 to 2004 | 1;2 | Armstrong & Conte, 2010 |
| Área de Proteção Ambiental Jundiaí | APJ | 23.241 | 46.958 | 30 | 52 | 1999 to 2005 | 1;2 | Ribeiro et al. 2005 |
| Estação Ecológica Jureia-Itatins | EEJ | 24.516 | 47.266 | 25 | * | * | 1;2 | Pombal & Gordo 2004 |
| Reserva Florestal Morro Grande | FMG | 23.726 | 46.968 | 25 | 32 | 2001 to 2004 | 3;4 | Dixo & Verdade 2006 |
| Parque Estadual Intervales | PEI | 24.174 | 47.952 | 25 | 162 | 1990 to 1991 | 1 | Bertoluci & Rodrigues 2002 |
| Estação Ambiental de Peti | AEP | 19.867 | 43.358 | 24 | 78 | 2002 to 2007 | 1 | Bertoluci et al. 2009 |
| Monte Verde | SMV | 21.483 | 41.866 | 19 | 5 | 2006 | 1;2;3;4 | Almeida-Gomes et al. 2010 |
| Parque Nacional da Serra da Bodoquena | PSB | 20.981 | 56.715 | 36 | 24 | 2005 | 1;2 | Uetanabaro et al. 2007 |
| Parque Estadual Morro do Diabo | PMD | 22.54 | 52.311 | 28 | 72 | 2005 to 2007 | 1;2;3;4 | Santos et al. 2009 |
| Nova Itapirema | NIT | 21.066 | 49.533 | 26 | 25 | 2003 to 2004 | 1;2 | Vasconcelos & Rossa-Feres 2005 |
| Icém | ICE | 20.333 | 49.183 | 25 | 19 | 2004 to 2005 | 1;2;4 | Silva et al. 2011; da Silva et al. 2012 |
| Parque Estadual Mata Godoy | PMG | 23.453 | 51.239 | 23 | 45 | 1995 to 1997 | 1;2 | Machado et al. 1999 |
| Estação Ecológica de Caetetus | EEC | 22.402 | 49.697 | 24 | 12 | 2005 to 2006 | 1;3 | Bertoluci et al. 2007 |
| Floresta Edmundo Navarro | FEN | 22.407 | 47.528 | 20 | 52 | 2001 to 2002 | 1;2;3 | Toledo et al. 2003 |
| Santa Fé do Sul | SFS | 20.183 | 50.883 | 19 | 18 | 2003 to 2004 | 1;2;4 | Santos et al. 2007 |
| União Paulista, Fazenda Boa Vista | FBV | 20.921 | 49.926 | 24 | 12 | 2006 to 2009 | 1;2;4 | Rossa-Feres et al. 2012 |
| São João de Iracema, Fazenda São Francisco | FSF | 20.473 | 50.293 | 24 | 12 | 2006 to 2009 | 1;2;4 | Rossa-Feres et al. 2012 |
| Distrito de Ida Iolanda, Fazenda Pauã | IFP | 20.742 | 49.929 | 24 | 12 | 2006 to 2009 | 1;2;4 | Rossa-Feres et al. 2012 |
| Pindorama, Estação Experimental | PEE | 21.22 | 48.917 | 22 | 12 | 2006 to 2009 | 1;2;4 | Rossa-Feres et al. 2012 |
| Matão, Fazenda Cambuhy | MFC | 21.62 | 48.537 | 21 | 24 | 2006 to 2009 | 1;2;4 | Rossa-Feres et al. 2012 |
| Votuporanga, Fazenda Primavera | VFP | 20.514 | 50.086 | 21 | 12 | 2006 to 2009 | 1;2;4 | Rossa-Feres et al. 2012 |
| Turmalina, Fazenda São João | TSJ | 20.003 | 50.433 | 21 | 24 | 2006 to 2009 | 1;2;4 | Rossa-Feres et al. 2012 |
| Nova Granada, Fazenda São João | GSJ | 20.543 | 49.246 | 20 | 12 | 2006 to 2009 | 1;2;4 | Rossa-Feres et al. 2012 |
| Sales, Fazenda Águas Claras | FAC | 21.404 | 49.5 | 20 | 12 | 2006 to 2009 | 1;2;4 | Rossa-Feres et al. 2012 |
| Planalto, Fazenda Taperão | PFT | 21.001 | 49.973 | 20 | 12 | 2006 to 2009 | 1;2;4 | Rossa-Feres et al. 2012 |
| Palestina, Fazenda Boa Vista | PBV | 20.321 | 49.504 | 20 | 12 | 2006 to 2009 | 1;2;4 | Rossa-Feres et al. 2012 |
| Novo Horizonte, Fazenda Serrinha | NHS | 21.52 | 49.294 | 18 | 12 | 2006 to 2009 | 1;2;4 | Rossa-Feres et al. 2012 |
| Santo Antonio do Aracanguá | SAA | 20.926 | 50.348 | 15 | 12 | 2006 to 2009 | 1;2;4 | Rossa-Feres et al. 2012 |
| Bebedouro, Fazenda Córrego dos Bois | FCB | 20.885 | 48.54 | 13 | 12 | 2006 to 2009 | 1;2;4 | Rossa-Feres et al. 2012 |
| Barretos, Fazenda Floresta | BFF | 20.637 | 48.751 | 16 | 12 | 2006 to 2009 | 1;2;4 | Rossa-Feres et al. 2012 |
| Taquaritinga, Fazenda Santa Lúcia | TSS | 21.402 | 48.687 | 9 | 12 | 2006 to 2009 | 1;2;4 | Rossa-Feres et al. 2012 |

* Data not informed in the consulted material. These references are books that provided species composition from the area studied.

# Pitfall traps used only in 2002.

APPENDIX S2

S2. Partition of the variance of Compositional Beta Diversity components based on Carvalho’s *et al.* (2012, β_cc_, β_3_ and β_rich_) approach explained by geographical distance (Space), current environmental gradients (Environ) and long-term climatic conditions (refugia) in 44 sites in Brazilian Atlantic Forest. Res = unexplained variance. “-” = variation explained < 0.


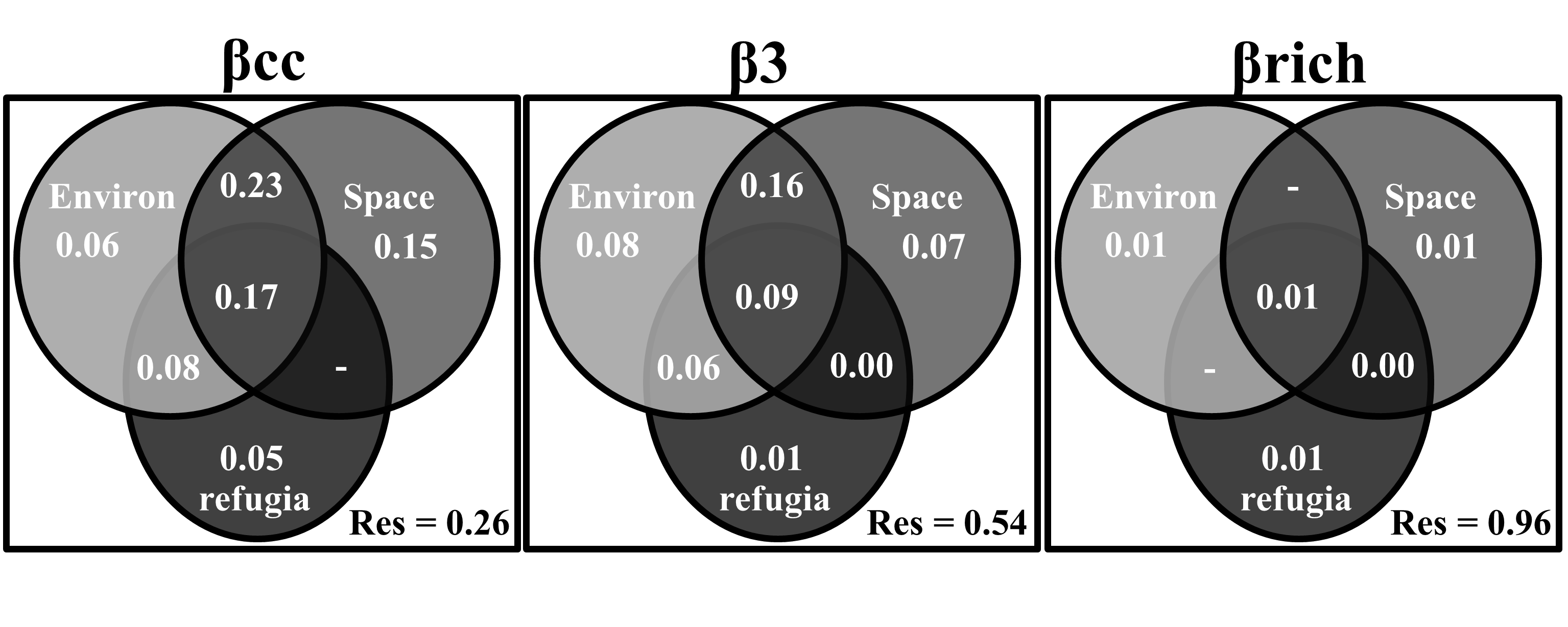


Although Baselga’s (2010, 2012) and Carvalho’s et al. (2012) approaches are intended to measure species replacement and species richness differences, their methods can lead to radically different conclusions using the same dataset (see Carvalho *et al.* 2013 for comparisons of these methods). The main difference observed in our study was total variation explaining turnover components (β_jtu_ and β_-3_). The total variation explained for β_jtu_ was 75% whereas total variance explained for β_-3_ was 45%. β_jtu_ overestimates replacement because it measures replacement relative to the species-poorer site and not as a proportion of all species (Carvalho *et al.*, 2013). Thus, β_jtu_ overlooks the fact that beta diversity is not always identical to species replacement if nestedness is lacking (Carvalho *et al*., 2013). The conceptual difference between β_jne_ and β_rich_ is that β_jne_ only incorporate differences in richness if sites are nested, whereas β_rich_ reflects all richness differences whether or not sites are nested (Carvalho *et al*., 2012, 2013). However, the outcome of the current debate surrounding Baselga’s (2010, 2012) and Carvalho’s *et al.* (2012) analyses is not over, and at this point, no consensus has been reached (see Leprieur & Oikonomou 2013).

APPENDIX S3

S3. Cladogram of anuran demonstrating the phylogenetic relationships of our data-set based

on the phylogenetic hypotheses proposed by Pyron & Wiens (2011). Red lines represent anuran species occurring only in stable region. Blue lines represent anuran species occurring only in unstable region. Black lines represent anuran species occurring in both stable and unstable regions.


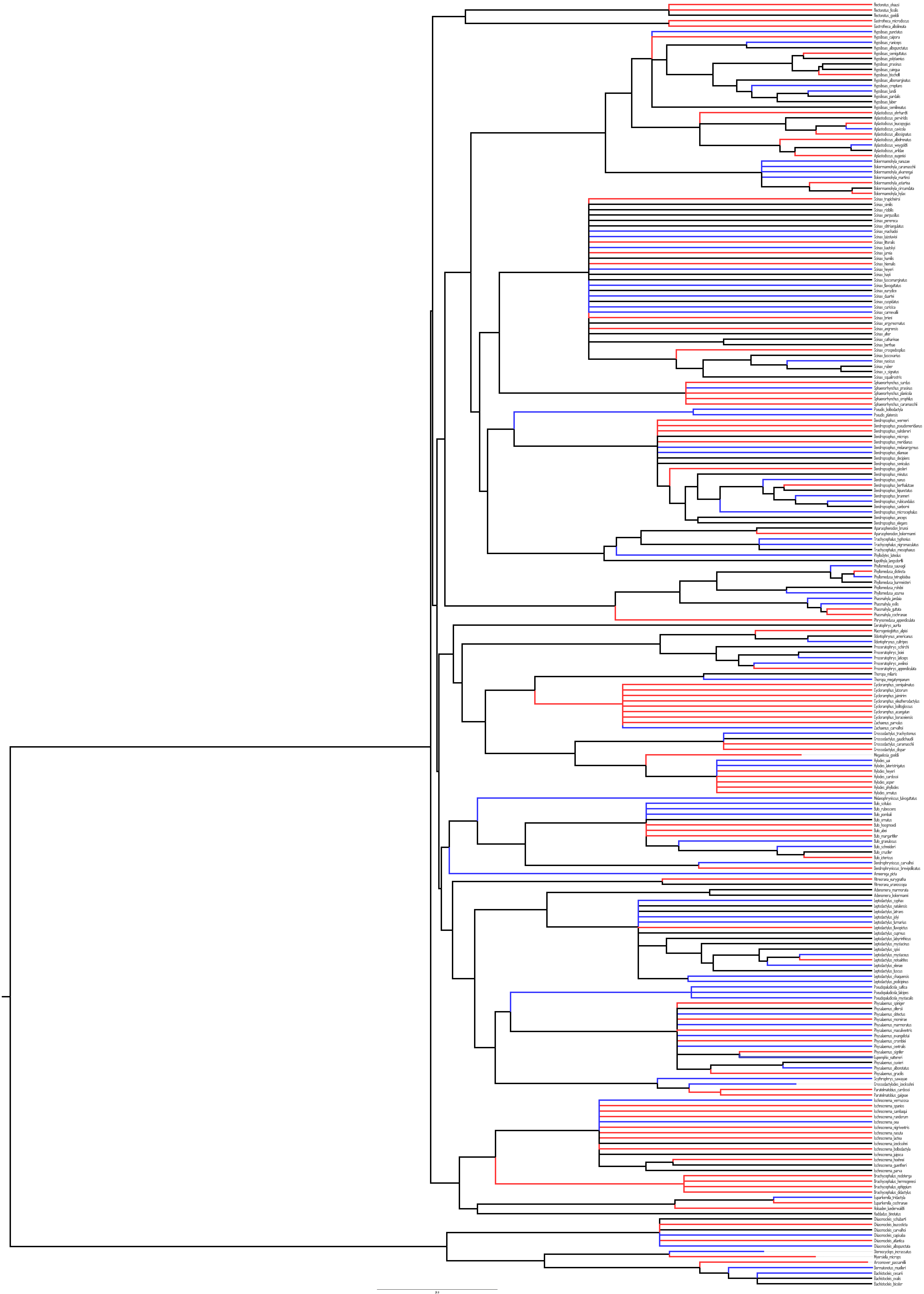


APPENDIX S4

S4. Taxonomic classification and occurrence of amphibian species in stable and unstable sites from Brazilian Atlantic Forest. We followed the nomenclature as given in Pyron & Wiens (2011). * = Anuran species present in the phylogeny proposed by Pyron & Wiens (2011).

| Family | Genera | Species | Stable sites | Unstable sites |
| --- | --- | --- | --- | --- |
| Brachycephalidae | *Brachycephalus** | *B. didactylus* | X | -- |
|  |  | *B. ephippium** | X | -- |
|  |  | *B. hermogenesi* | X | -- |
|  |  | *B. nodoterga* | X | -- |
|  | *Ischnocnema** | *I. bolbodactyla* | X | -- |
|  |  | *I. guentheri** | X | X |
|  |  | *I. hoehnei** | X | -- |
|  |  | *I. izecksohni* | X | X |
|  |  | *I. juipoca** | X | X |
|  |  | *I. lactea* | X | -- |
|  |  | *I. nasuta* | X | -- |
|  |  | *I. nigriventris* | X | -- |
|  |  | *I. oea* | -- | X |
|  |  | *I. parva** | X | X |
|  |  | *I. randorum* | X | -- |
|  |  | *I. sambaqui* | X | -- |
|  |  | *I. spanios* | X | -- |
|  |  | *I. verrucosa* | -- | X |
| Bufonidae | *Melanophryniscus** | *M. fulvoguttatus* | -- | X |
|  | *Dendrophryniscus** | *D. brevipollicatus* | X | -- |
|  |  | *D. carvalhoi* | -- | X |
|  | *Bufo** | *B. abei* | X | -- |
|  |  | *B. crucifer** | X | X |
|  |  | *B. granulosus** | -- | X |
|  |  | *B. hoogmoedi* | X | -- |
|  |  | *B. ictericus** | X | -- |
|  |  | *B. margaritifer** | X | -- |
|  |  | *B. ornatus* | X | X |
|  |  | *B. pombali* | -- | X |
|  |  | *B. rubescens* | -- | X |
|  |  | *B. schneideri** | -- | X |
|  |  | *B. scitulus* | -- | X |
| Centrolenidae | *Vitreorana** | *V. eurygnatha** | X | -- |
|  |  | *V. uranoscopa* | X | X |
| Ceratophryidae | *Ceratophrys** | *C. aurita* | X | X |
| Craugastoridae | *Haddadus ** | *H. binotatus** | X | X |
|  | *Euparkerella* | *E. cochranae* | X | -- |
|  |  | *E. tridactyla* | -- | X |
| Craugastoridae | *Holoaden ** | *H. luederwaldti** | X | -- |
| Cycloramphidae | *Crossodactylodes* | *C. izecksohni* | -- | X |
|  | *Cycloramphus** | *C. acangatan** | X | -- |
|  |  | *C. bolitoglossus* | X | -- |
|  |  | *C. boraceiensis** | X | -- |
|  |  | *C. eleutherodactylus* | X | -- |
|  |  | *C. juimirim* | X | -- |
|  |  | *C. lutzorum* | X | -- |
|  |  | *C. semipalmatus* | X | -- |
|  | *Macrogenioglottus** | *M. alipioi** | X | -- |
|  | *Odontophrynus ** | *O. americanus** | X | X |
|  |  | *O. cultripes** | -- | X |
|  | *Proceratophrys** | *P. appendiculata** | X | -- |
|  |  | *P. avelinoi** | -- | X |
|  |  | *P. boiei** | X | X |
|  |  | *P. laticeps** | -- | X |
|  |  | *P. schirchi** | X | X |
|  | *Thoropa** | *T. megatympanum* | -- | X |
|  |  | *T. miliaris** | X | X |
| Cycloramphidae | *Zachaenus* | *Z. carvalhoi* | -- | X |
|  |  | *Z. parvulus* | X | -- |
| Dendrobatidae | *Ameerega** | *A. picta** | -- | X |
| Hemiphractidae | *Flectonotus** | *F. goeldii* | X | X |
|  |  | *F. fissilis* | X | -- |
|  |  | *F. ohausi* | X | -- |
|  | *Gastrotheca** | *G. albolineata* | X | -- |
|  |  | *G. microdiscus* | X | -- |
| Hylidae | *Aparasphenodon** | *A. bokermanni* | X | -- |
|  |  | *A. brunoi** | X | X |
|  | *Aplastodiscus** | *A. albofrenatus** | X | -- |
|  |  | *A. albosignatus** | X | -- |
|  |  | *A. arildae** | X | X |
|  |  | *A. cavicola** | -- | X |
|  |  | *A. ehrhardti* | X | -- |
|  |  | *A. eugenioi** | X | -- |
|  |  | *A. leucopygius** | X | -- |
|  |  | *A. perviridis** | X | X |
|  |  | *A. weygoldti** | -- | X |
|  | *Bokermannohyla ** | *B. alvarengai* | -- | X |
|  |  | *B. astartea** | X | -- |
|  |  | *B. caramaschii* | -- | X |
|  |  | *B. circumdata** | X | X |
|  |  | *B. hylax** | X | -- |
|  |  | *B. martinsi** | -- | X |
|  |  | *B. nanuzae* | -- | X |
|  | *Dendropsophus** | *D. anceps** | X | X |
| Hylidae | *Dendropsophus** | *D. berthalutzae** | X | -- |
|  |  | *D. bipunctatus** | X | X |
|  |  | *D. branneri** | -- | X |
|  |  | *D. decipiens* | X | X |
|  |  | *D. elegans** | X | X |
|  |  | *D. elianeae* | -- | X |
|  |  | *D. giesleri** | X | -- |
|  |  | *D. melanargyreus* | -- | X |
|  |  | *D. meridianus* | X | -- |
|  |  | *D. microcephalus** | -- | X |
|  |  | *D. microps* | X | X |
|  |  | *D. minutus** | X | X |
|  |  | *D. nahdereri* | X | -- |
|  |  | *D. nanus** | -- | X |
|  |  | *D. pseudomeridianus* | X | -- |
|  |  | *D. rubicundulus** | -- | X |
|  |  | *D. sanborni** | X | X |
|  |  | *D. seniculus** | X | X |
|  |  | *D. werneri* | X | -- |
|  | *Hypsiboas** | *H. albomarginatus** | X | X |
|  |  | *H. albopunctatus** | X | X |
|  |  | *H. bischoffi** | X | -- |
|  |  | *H. caingua** | X | X |
|  |  | *H. caipora* | X | -- |
|  |  | *H. crepitans** | -- | X |
|  |  | *H. faber** | X | X |
|  |  | *H. lundii** | -- | X |
|  |  | *H. pardalis** | X | X |
|  |  | *H. polytaenius** | X | X |
|  |  | *H. prasinus** | X | X |
|  |  | *H. punctatus* | -- | X |
|  |  | *H. raniceps** | -- | X |
|  |  | *H. semiguttatus** | X | -- |
|  |  | *H. semilineatus** | X | X |
|  | *Itapotihyla** | *I. langsdorffii** | X | X |
|  | *Phyllodytes** | *P. luteolus** | -- | X |
|  | *Pseudis** | *P. bolbodactyla** | -- | X |
|  |  | *P. platensis* | -- | X |
|  | *Scinax** | *S. alter* | X | X |
|  |  | *S. angrensis* | X | -- |
|  |  | *S. argyreornatus* | X | X |
|  |  | *S. berthae** | X | X |
|  |  | *S. brieni* | X | -- |
|  |  | *S. carnevallii* | -- | X |
|  |  | *S. catharinae** | X | X |
|  |  | *S. crospedospilus** | X | -- |
| Hylidae | *Scinax** | *S. curicica* | -- | X |
|  |  | *S. cuspidatus* | X | X |
|  |  | *S. duartei* | -- | X |
|  |  | *S. eurydice* | X | X |
|  |  | *S. flavoguttatus* | -- | X |
|  |  | *S. fuscomarginatus* | X | X |
|  |  | *S. fuscovarius** | X | X |
|  |  | *S. hayii* | X | X |
|  |  | *S. heyeri* | -- | X |
|  |  | *S. hiemalis* | X | -- |
|  |  | *S. humilis* | X | X |
|  |  | *S. jureia* | X | -- |
|  |  | *S. kautskyi* | -- | X |
|  |  | *S. littoralis* | X | -- |
|  |  | *S. luizotavioi* | -- | X |
|  |  | *S. machodoi* | -- | X |
|  |  | *S. nasicus** | -- | X |
|  |  | *S. obtriangulatus* | X | X |
|  |  | *S. perereca* | X | X |
|  |  | *S. perpusillus* | X | X |
|  |  | *S. rizibilis* | X | X |
|  |  | *S. ruber** | X | X |
|  |  | *S. similis* | X | X |
|  |  | *S. squalirostris** | X | X |
|  |  | *S. trapicheiroi* | X | -- |
|  |  | *S. x-signatus** | X | X |
|  | *Sphaenorhynchus** | *S. caramaschii* | X | -- |
|  |  | *S. orophilus** | X | -- |
|  |  | *S. planicola* | X | -- |
|  |  | *S. prasinus* | -- | X |
|  |  | *S. surdus* | X | -- |
|  | *Trachycephalus** | *T. mesophaeus** | X | X |
|  |  | *T. nigromaculatus** | -- | X |
|  |  | *T. typhonius* | -- | X |
|  | *Phasmahyla** | *P. cochranae** | X | -- |
|  |  | *P. exilis** | -- | X |
|  |  | *P. guttata** | X | -- |
|  |  | *P. jandaia** | -- | X |
|  | *Phrynomedusa ** | *P. appendiculata* | X | -- |
|  | *Phyllomedusa** | *P. azurea** | -- | X |
|  |  | *P. burmeisteri** | X | X |
|  |  | *P. distincta** | X | -- |
|  |  | *P. rohdei** | X | X |
|  |  | *P. sauvagii** | -- | X |
|  |  | *P. tetraploidea** | -- | X |
| Hylodidae | *Crossodactylus** | *C. caramaschii** | X | -- |
| Hylodidae | *Crossodactylus** | *C. dispar* | X | -- |
|  |  | *C. gaudichaudii* | X | X |
|  |  | *C. trachystomus* | -- | X |
| Hylodidae | *Hylodes** | *H. asper* | X | -- |
|  |  | *H. cardosoi* | X | -- |
|  |  | *H. heyeri* | X | -- |
|  |  | *H. lateristrigatus* | -- | X |
|  |  | *H. ornatus** | X | -- |
|  |  | *H. phyllodes** | X | -- |
|  |  | *H. uai* | -- | X |
|  | *Megaloesia* | *M. goeldii* | X | -- |
| Leiuperidae | *Eupemphix** | *E. nattereri** | -- | X |
|  | *Physalaemus** | *P. albonotatus** | -- | X |
|  |  | *P. centralis* | -- | X |
|  |  | *P. crombiei* | X | -- |
|  |  | *P. cuvieri** | X | X |
|  |  | *P. evangelistai* | -- | X |
|  |  | *P. gracilis ** | X | -- |
|  |  | *P. maculiventris* | X | -- |
|  |  | *P. marmoratus* | -- | X |
|  |  | *P. moreirae* | X | -- |
|  |  | *P. obtectus* | -- | X |
|  |  | *P. olfersii* | X | X |
|  |  | *P. signifer** | X | -- |
|  |  | *P. spiniger* | X | -- |
|  | *Pseudopaludicola** | *P. falcipes** | -- | X |
|  |  | *P. mystacalis* | -- | X |
|  |  | *P. saltica* | -- | X |
| Leptodactylidae | *Adenomera** | *A. bokermanni* | X | X |
|  |  | *A. marmorata* | X | X |
|  | *Leptodactylus** | *L. chaquensis** | -- | X |
|  |  | *L. cupreus* | X | X |
|  |  | *L. elenae** | -- | X |
|  |  | *L. flavopictus* | X | -- |
|  |  | *L. furnarius* | -- | X |
|  |  | *L. fuscus** | X | X |
|  |  | *L. jolyi* | -- | X |
|  |  | *L. labyrinthicus** | X | X |
|  |  | *L. latrans* | X | X |
|  |  | *L. mystaceus** | -- | X |
|  |  | *L. mystacinus** | X | X |
|  |  | *L. natalensis* | -- | X |
|  |  | *L. notoaktites** | X | -- |
|  |  | *L. podicipinus** | -- | X |
|  |  | *L. spixi** | X | X |
|  |  | *L. syphax* | -- | X |
| Leptodactylidae | *Paratelmatobius ** | *P. cardosoi** | X | -- |
|  |  | *P. gaigeae** | X | -- |
|  | *Scythrophrys** | *S. sawayae** | X | -- |
| Microhylidae | *Arcovomer* | *A. passarellii* | X | -- |
|  | *Myersiella* | *M. microps* | X | -- |
|  | *Stereocyclops* | *S. incrassatus* | -- | X |
|  | *Chiasmocleis ** | *C. albopunctata* | -- | X |
|  |  | *C. atlantica* | X | -- |
|  |  | *C. capixaba* | -- | X |
|  |  | *C. carvalhoi* | X | X |
|  |  | *C. leucosticta* | X | -- |
|  |  | *C. schubarti* | X | X |
|  | *Dermatonotus ** | *D. muelleri** | -- | X |
|  | *Elachistocleis ** | *E. bicolor* | X | X |
|  |  | *E. cesarii* | -- | X |
|  |  | *E. ovalis** | X | X |

APPENDIX S5

S5. Analysis summary of the principal component analysis (PCA) for 44 sites in the Brazilian Atlantic Forest. PPT = annual precipitation; PPTS = precipitation seasonality (coefﬁcient of variation across months); PPTW = precipitation of wettest quarter; PPTD = precipitation of driest quarter; and DIF.P = difference between PPTW and PPTD; ANNT = annual mean temperature; MAXT = maximum temperature of the warmest month; MINT = minimum temperature of the coldest month; DIF.T = difference between MAXT and MINT; MAEL = maximum elevation; MIEL = minimum elevation; DIEL = difference between MAEL and MIEL.

|  | **PC1** | **PC2** | **PC3** |
| --- | --- | --- | --- |
| ANNT | 0.39891 | -0.02463 | -0.21675 |
| MAXT | 0.392707 | -0.0519 | -0.19697 |
| MINT | 0.299237 | -0.18214 | -0.40346 |
| DIF.T | 0.209691 | 0.195294 | 0.291103 |
| MAEL | -0.30688 | 0.286701 | -0.05176 |
| MIEL | -0.09172 | 0.268362 | 0.539654 |
| DIEL | -0.23857 | 0.101078 | -0.41037 |
| PPT | -0.37093 | -0.03795 | -0.22817 |
| PPTW | -0.2235 | 0.394018 | -0.30626 |
| PPTD | -0.35929 | -0.29922 | -0.06909 |
| PPTS | 0.274458 | 0.452125 | -0.03379 |
| DIF.P | 0.041042 | 0.557059 | -0.2294 |





**REFERENCES**

Almeida-Gomes, M., Almeida-Santos, M., Goyannes-Araújo, P., Borges-Júnior, V.N.T., Vrcibradic, D., Siqueira, C.C., Ariani, C.V., Dias, A.S., Souza, V.V., Pinto, R.R., Van Sluys, M. & Rocha, C.F.D. (2010) Anurofauna of an Atlantic Rainforest fragment and its surroundings in Northern Rio de Janeiro State, Brazil. *Brazilian Journal Bioliology*, **70**, 871-877. Available from: <http://www.scielo.br/pdf/bjb/v70n3s0/18.pdf>

Armstrong, C.G. & Conte, C.E. (2010) Assemblage of anurans (Amphibia, Anura) of an area of Atlantic Forest, South of Brazil. *Biota Neotropica*, **10**, Available from: <http://www.biotaneotropica.org.br/v10n1/en/abstract?article+bn00610012010>

Araujo, C.O., Condez, T.H., Bovo, R.P., Centeno, F.C. & Luiz, A.M. (2010) Amphibians and reptiles of the Parque Estadual Turístico do Alto Ribeira (PETAR), SP: an Atlantic Forest remnant of Southeastern Brazil. *Biota Neotropica*, **10**, 257-274. Available from: <http://www.scielo.br/pdf/bn/v10n4/31.pdf>

Baselga, A. (2010) Partitioning the turnover and nestedness components of beta diversity. *Global Ecology and Biogeography*, **19**, 134–143.

Baselga, A. (2012) The relationship between species replacement, dissimilarity derived from nestedness, and nestedness. *Global Ecology and Biogeography*, **21**, 1223–1232.

Bertoluci, J., Brassaloti, R.A., Ribeiro Jr, J.W., Vilela, V.M.F.N. & Sawakuchi, H.O. (2007) Species composition and similarities among anuran assemblages of forest sites in southeastern *Brazilian Scientia Agricola*, **64**, 364-374. Available from: <http://www.scielo.br/pdf/sa/v64n4/06.pdf>

Bertoluci, J., Canelas, M.A.S., Eisemberg, C.C., Palmuti C.F.S. & Montingelli G.G. (2009) Herpetofauna of Estação Ambiental de Peti, an Atlantic Rainforest fragment of Minas Gerais State, southeastern Brazil. *Biota Neotropica*, **9**, Available from: <http://www.biotaneotropica.org.br/v9n1/en/abstract?inventory+bn01409012009>

Bertoluci, J. & Rodrigues, M.T. (2002) Utilização de hábitats reprodutivos e micro-hábitats de vocalização em uma taxocenose de anuros (Amphibia) da Mata Atlântica do sudeste do Brasil. *Papéis Avulsos de Zoologia*, **42**, 287-297. Available from: <http://www.scielo.br/pdf/paz/v42n11/17500.pdf>

Canelas, M.A.S & Bertoluci, J. (2007) Anurans of the Serra do Caraça, southeastern Brazil: species composition and phenological patterns of calling activity. *Iheringia*, **97**, 21-26. Available from: <http://www.scielo.br/pdf/isz/v97n1/a04v97n1.pdf>

Carvalho, J.C., Cardoso, P. & Gomes, P. (2012) Determining the relative roles of species replacement and species richness differences in generating beta diversity patterns. *Global Ecology and Biogeography*, **21**, 760–771.

Carvalho, J.C., Cardoso, P., Borges, P.A.V., Schmera, D. & Podani, J. (2013) Measuring fractions of beta diversity and their relationships to nestedness: a theoretical and empirical comparison of novel approaches. *Oikos*, **122**, 825–834.

Carvalho-e-Silva, A.M.T., Silva, G.R., Carvalho-e-Silva, S.P. (2008) Anurans at Rio das Pedras Reserve, Mangaratiba, RJ, Brazil. [Internet] *Biota Neotropica,* **8**, Available from: <http://www.biotaneotropica.org.br/v8n1/en/abstract?inventory+bn02608012008>

Condez, T.H, Sawaya, R.J. & Dixo, M. (2009) Herpetofauna of the Atlantic Forest remnants of Tapiraí and Piedade region, São Paulo state, southeastern Brazil. *Biota Neotropica*, **9**, Available from: <http://www.biotaneotropica.org.br/v9n1/en/abstract?inventory+bn01809012009>

Conte, C.E. & Rossa-Feres, D.C. (2006) Diversidade e ocorrência temporal da anurofauna (Amphibia, Anura) em São José dos Pinhas, Paraná, Brasil. *Revista Brasileira de Zoologa*, **23**, 162-175. Available from: <http://www.scielo.br/pdf/rbzool/v23n1/a08v23n1.pdf>

da Silva, F.R., Candeira, C.P. & Rossa-Feres, D.C. (2012) Dependence of anuran diversity on environmental descriptors in farmland ponds. *Biodiversity and Conservation*, **21**, 1411-1424. Available from: <http://link.springer.com/article/10.1007%2Fs10531-012-0252-z?LI=true>

Dixo, M. & Verdade, V.K. (2006) Leaf litter herpetofauna of the Reserva Florestal de Morro Grande, Cotia (SP). *Biota Neotropica*, **6**, Available from: <http://www.biotaneotropica.org.br/v6n2/pt/abstract?article+bn00806022006>

Feio, R.N., Braga, U.M.L., Wiederhecker, H. & Santos, P.S. (1998) *Anfíbios do Parque Estadual do Rio Doce (Minas Gerais)*. Universidade Federal de Viçosa, Instituto Estadual de Florestas, MG.

Forlani, M.C., Bernardo, P.H., Haddad, C.F.B. & Zaher, H. (2010) Herpetofauna do Parque Estadual Carlos Botelho, São Paulo, Brasil. *Biota Neotropica*, **10**, Available from:

<http://www.biotaneotropica.org.br/v10n3/en/abstract?inventory+bn00210032010>

Hartmann, M.T. (2004) Biologia reprodutiva de uma comunidade de anuros (Amphibia) na mata atlântica (Picinguaba, Ubatuba, SP). Tese de Doutorado. Insituto de Biociências, Universidade Estadual Paulista, Rio Claro, SP.

Heyer, W.R., Rand, A.S., Cruz, C.A.G.da, Peixoto, O.L. & Nelson, C.E. (1990) Frogs of Boracéia. *Arquivos de Zoologia*, **31**, 231-410.

Leprieur, F. & Oikonomou, A. (2013) The need for richness independent measures of turnover when delineating biogeographical regions. *Journal of Biogeography*, **41**, 417-420.

Machado, R.A., Bernarde, P.S., Morato, S.A.A. & Anjos, L. (1999) Análise comparada da riqueza de anuros entre duas áreas com diferentes estados de conservação no Município de Londrina, Paraná, Brasil (Amphibia, Anura). *Revista Brasileira de Zoologia*, **16**, 997-1004. Available from: <http://www.scielo.br/pdf/rbzool/v16n4/v16n4a09.pdf>

Pombal JR., J.P. & Gordo, M. (2004) Anfíbios Anuros da Juréia. In *Estação Ecológica Juréia-Itatins. Ambiente Físico, Flora e Fauna* (eds. O.A.V. Marques & W. Duleba), pp. 243-256. Holos Editora, Ribeirão Preto.

Pyron, R.A. & Wiens, J.J. (2011) A large-scale phylogeny of Amphibia including over 2800 species, and a revised classification of extant frogs, salamanders, and caecilians. *Molecular Phylogenetics and Evolution*, **61**, 543–583.

Ramos, A.D. & Gasparini, J.L. (2004) *Anfíbios do Goiapaba-Açu, Fundão, Estado do Espírito Santo*. 75p.

Ribeiro, R.S., Egito, G.T.B.T., & Haddad, C.F.B. (2005) Chave de identificação: anfíbios anuros da vertente de Jundiaí da Serra do Japi, estado de São Paulo. *Biota Neotropica*, **5**, 1-15, Available from: <http://www.scielo.br/pdf/bn/v5n2/v5n2a16.pdf>

Rossa-Feres, D.C., Prado, V.H.M., Silva, F.R. & Almeida, H.J. (2012) Diversidade de anuros em fragmentos florestais na região noroeste do estado de São Paulo. *In*: Orlando Necchi Junior (Org). Fauna e flora de fragmentos florestais remanescentes da região noroeste do estado de São Paulo. 1ed. Ribeirão Preto, Holos, p 207-222.

Santos, T.G., Rossa-Feres, D.C. & Casatti, L. (2007) Diversidade e distribuição espaço-temporal de anuros em região com pronunciada estação seca no sudeste do Brasil. *Iheringia*, **97**, 37-49, Available from: <http://www.scielo.br/pdf/isz/v97n1/a07v97n1.pdf>

Santos, T.G., Vasconcelos, T.S., Rossa-Feres, D.C. & Haddad, C.F.B. (2009) Anurans of a seasonally dry tropical forest: Morro do Diabo State Park, São Paulo state, Brazil. *Journal of Natural History* **43**, 973-993. Available from: <http://www.tandfonline.com/doi/abs/10.1080/00222930802702498>

Serafim, H., Cicchi, P.J.P., Ienne, S. & Jim, J. (2008) Anurans of remnants of Atlantic forest of São José do Barreiro municipality, São Paulo State, Brazil. *Biota Neotropica*, **8**, Available from: <http://www.biotaneotropica.org.br/v8n2/en/abstract?article+bn01008022008>

Silva, R.A., Martins, I.A. & Rossa-Feres, D.C. (2011) Environmental heterogeneity: anuran diversity in homogeneous envionments. *Zoologia*, **28**, 610-618, Available from: <http://www.scielo.br/pdf/zool/v28n5/a09v28n5.pdf>

Silva-Soares, T., Hepp, F., Costa, P.N., Luna-Dias, C., Gomes, M.R., Carvalho-e-Silva, A.M.P.T. & Carvalho-e-Silva, S.P. (2010) Anuran Amphibians from RPPN Campo Escoteiro Geraldo Hugo Nunes, Guapimirim Municipality, Rio de Janeiro, Southeastern Brazil. *Biota Neotropica*, **10**, Available from: <http://www.biotaneotropica.org.br/v10n2/en/abstract?inventory+bn01210022010>

Toledo, L.F., Zina, J. & Haddad, C.F.B. (2003) Distribuição espacial e temporal de uma comunidade de anfíbios anuros do município de Rio Claro, São Paulo, Brasil. *Holos Environmental*, **3**, 136-149. Available from: <http://www.periodicos.rc.biblioteca.unesp.br/index.php/holos/article/view/1126/1038>

Tonini, J.F.R., Carão, L.M., Pinto, I.S., Gasparini, J.L., Leite, Y.L.R. & Costa, L.P. (2010) Non-volant tetrapods from Reserva Biológica de Duas Bocas, State of Espírito Santo, Southeastern Brazil. *Biota Neotropica*, **10**, Available from: <http://www.biotaneotropica.org.br/v10n3/en/abstract?inventory+bn02710032010>

Uetanabaro M., Souza F.L., Filho P.L., Beda A.F., Brandão R.A. (2007) Anfíbios e répteis do Parque Nacional da Serra da Bodoquena, Mato Grosso do Sul, Brasil [Internet]. *Biota Neotropica*, **7**, Available from: <http://www.biotaneotropica.org.br/v7n3/pt/abstract?article+bn01207032007>

Vasconcelos, T.S. & Rossa-Feres, D.C. (2005) Diversidade, distribuição espacial e temporal de anfíbios anuros (Amphibia, Anura) na região noroeste do estado de São Paulo, Brasil. *Biota Neotropica,* **5**, Available from: [www.biotaneotropica.org.br/v5n2/pt/abstract?article+BN01705022005](http://www.biotaneotropica.org.br/v5n2/pt/abstract?article+BN01705022005)
